# Is there a benefit for anesthesiologists of adding difficult airway scenarios for learning fiberoptic intubation skills using virtual reality training? A randomized controlled study

**DOI:** 10.1371/journal.pone.0281016

**Published:** 2023-01-27

**Authors:** Loic Cailleau, Thomas Geeraerts, Vincent Minville, Olivier Fourcade, Thomas Fernandez, Jean Etienne Bazin, Linden Baxter, Vassilis Athanassoglou, Henry Jefferson, Anika Sud, Tim Davies, Cyprian Mendonca, Matteo Parotto, Matt Kurrek

**Affiliations:** 1 Department of Anesthesia and Intensive Care, University Toulouse 3 Paul Sabatier, Toulouse, France; 2 Department of Anesthesia and Intensive Care, University Clermont Auvergne, Clermont Ferrand, France; 3 Department of Anesthesia, Oxford University, Oxford, United Kingdom; 4 Department of Anesthesia, University of Warwick and Coventry, Coventry, United Kingdom; 5 Department of Anesthesia, University of Toronto, Toronto, Canada; Sapienza University of Rome: Universita degli Studi di Roma La Sapienza, ITALY

## Abstract

Fiberoptic intubation for a difficult airway requires significant experience. Traditionally only normal airways were available for high fidelity bronchoscopy simulators. It is not clear if training on difficult airways offers an advantage over training on normal airways. This study investigates the added value of difficult airway scenarios during virtual reality fiberoptic intubation training. A prospective multicentric randomized study was conducted 2019 to 2020, among 86 inexperienced anesthesia residents, fellows and staff. Two groups were compared: Group N (control, n = 43) first trained on a normal airway and Group D (n = 43) first trained on a normal, followed by three difficult airways. All were then tested by comparing their ORSIM^®^ scores on 5 scenarios (1 normal and 4 difficult airways). The final evaluation ORSIM^®^ score for the normal airway testing scenario was significantly higher for group N than group D: median score 76% (IQR 56.5–90) versus 58% (IQR 51.5–69, p = 0.0039), but there was no difference in ORSIM^®^ scores for the difficult intubation testing scenarios. A single exposure to each of 3 different difficult airway scenarios did not lead to better fiberoptic intubation skills on previously unseen difficult airways, when compared to multiple exposures to a normal airway scenario. This finding may be due to the learning curve of approximately 5–10 exposures to a specific airway scenario required to reach proficiency.

## Introduction

The management of difficult airways is associated with significant mortality in both the Intensive Care Unit (ICU) and the operating room [1, 2]. Airway management devices have evolved and guidelines in airway management have changed over the last decade [3–7]. Flexible bronchoscopy (FB) remains an accepted first choice for patients with known difficult intubation or as a rescue technique for non-anticipated difficult intubations [8]. However, learning FB remains challenging because of its infrequent use which is further compounded by increasing use of video-laryngoscopes [4, 8–11]. Simulation appears to be an efficient tool for training health care providers using otherwise rare clinical scenarios [12–14]. Many studies have indeed shown the value of simulation for bronchoscopy training but none of them have studied its use during difficult airway management [15–26].

Until the recent development of a virtual reality fiberoptic simulator with multiple difficult airway scenarios, mostly normal airways were available for high fidelity bronchoscopy simulators [15, 17, 21, 22, 27–29]. It is, however, not clear if training on difficult airway scenarios offers a significant advantage over training on normal airway scenarios [30].

We hypothesized that inexperienced anesthesia providers would improve by training on a virtual reality simulator with difficult airway scenarios when compared to training on normal airway scenarios (primary outcome: improved performance score, secondary outcome: success rates, times, collision avoidance and perceived level of difficulty).

## Materials and methods

This study recruited anesthesia residents, fellows and staff physicians who had performed 10 or less fiberoptic intubations from June 2019 to March 2020. Participating centers were: Toulouse (France), Clermont-Ferrand (France), Coventry (United Kingdom) and Oxford (United Kingdom). The study protocol was not registered but communicated to all participating centers.

### Consent

Each participating center obtained ethics approval through their local Research Ethics Committee. This study’s protocol has been registered with the Comité d’Ethique pour les Recherches (CER) of Toulouse University under the number: 2018–099 for the centers in Toulouse and Clermont-Ferrand (France) and by the Health and Research Authority in Wales (Ref 19/HRA/5142) for the centers in Oxford and Coventry (United Kingdom). All participants gave written informed consent.

### Virtual reality high fidelity simulator

The ORSIM^®^ virtual reality bronchoscopy simulator (Airway Simulation Limited, Auckland, New Zealand) incorporates a replica video bronchoscope, desktop sensor module, and dedicated laptop computer. The ORSIM^®^ contains software recreating a high-fidelity FB intubation scenario for the user. It includes learning modules on airway anatomy and dexterity and uses both normal and difficult airway intubation scenarios. (https://www.orsim.co.nz/design).

### ORSIM^®^ score

Validity and reliability of the ORSIM^®^ simulator as an assessment tool have been shown in a previous study by Baker *et al*. and relied on external scoring of a large number of video recording, which was not felt to be feasible for research [30]. Their results provided them the basis for establishing computer-generated metrics [30] and the development of a proprietary ORSIM^®^ score. This ORSIM^®^ Score (which has not been validated against expert scores) appears on the ORSIM^®^ Session Results screen after the participant finishes a given scenario and was recorded to grade their performance.

### Gopher-module

To assess pre-test dexterity of each participant, the ‘Gopher’ module, contained in the ORSIM^®^, was used: it consists of a challenge game to catch nine different gopher figures using the ORSIM^®^ bronchoscope.

### Data collection

Demographic data were collected including age, gender, dominant hand, years of residency training, previous FB experience, previous ORSIM^®^ and video game experience. The ORSIM^®^ simulator’s data were collected including type of scenario, time to successful completion, collision avoidance percentage score and minimal oxygen saturation, ORSIM^®^ scores for each scenario and the degree of difficulty felt for each of the scenarios as judged by the study subjects.

### Study design

**Fig 1:**
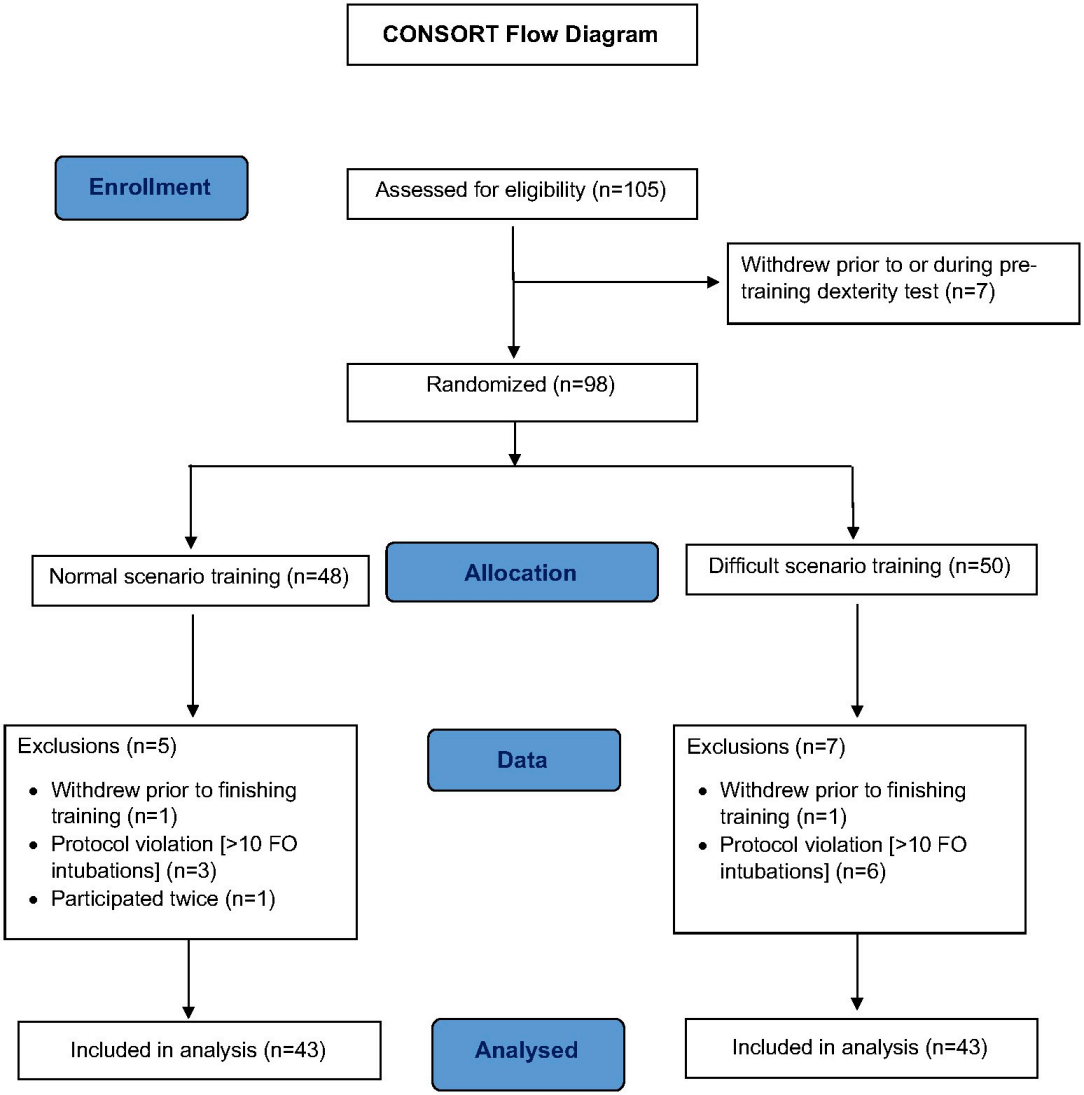
CONSORT flow diagram.

The participants recruitment, randomization and allocation are displayed in Fig 1. After obtaining written informed consent, a study form was completed. Participants then watched a video explaining the functionalities of the ORSIM^®^ simulator and the flexible bronchoscope. A pre-test dexterity test (Gopher module) was done before and then again after five minutes of hands-on familiarization with the bronchoscope simulator.

Afterwards participants were randomized (by random number drawing) into two groups: Group N trained on normal airway scenarios up to a maximum of 40 minutes and group D trained instead on one normal and then three difficult airway scenarios (a retropharyngeal abscess, an epiglottic cyst, and a macroglossia). Each of the participants in group D had up to 10 minutes per scenario before being prompted to move to the next scenario (i.e. up to a total of 40 minutes). Completion times, ORSIM^®^ scores, collision avoidance, lowest oxygen saturation, perceived level of difficulty on a Visual Analogic Scale (VAS) from 0 (easy) to 100 (very difficult) were recorded.

The final step consisted of testing the participants on their fiberoptic difficult intubation skills by using one normal and four difficult scenarios (airway trauma, severe epiglottitis, false cord cyst and angioneurotic oedema). Each participant had to try to succeed once in a maximum of ten minutes for each scenario. Times, ORSIM^®^ scores, collision avoidance, lowest oxygen saturation, perceived level of difficulty on a VAS from 0 (easy) to 100 (very difficult) were recorded. The investigator was not allowed to guide or help the participant to solve scenarios. The investigator’s role was strictly to observe and keep time, as well as manage any technical difficulty with the ORSIM^®^ simulator.

### Primary outcome

ORSIM^®^ scores for the final test scenarios were compared between the two groups for each scenario.

### Secondary outcomes

Times and ORSIM^®^ scores in normal airway scenarios before and after training were compared between the two groups. Successful rates, times, collision avoidance and perceived level of difficulty were also compared.

### Statistical analyses

Redcap^®^ software (Research Electronic Data Capture^®^) was used to collect data [31, 32]. R^®^ software (R Foundation for Statistical Computing, Vienna, Austria) was used for the statistical analyses. Power of this study had been estimated by using the data from the previous ORSIM^®^ study by Baker *et al*. [30]: 150 participants were needed to show a significant difference of 25% with a power of 90%. A Chi square test and Fisher’s exact test were used to test differences between group N and D concerning categorial variables. A T-test (or Wilcoxon-Mann-Whitney for not normally distributed data) was used to test differences concerning continuous or ordinal variables. Statistical significance was taken as p<0.05. Results are expressed in median and interquartile ranges or in numbers and percentages.

## Results

### Recruitment and exclusions

The study began in June 2019 and was stopped in March 2020 due to the Covid-19 pandemic. This analysis includes the 86 participants out of the 150 expected enrolments (43 in group N, 43 in group D) from Toulouse (n = 25) and Clermont-Ferrand (n = 25) in France, the University Hospitals Coventry and Warwickshire NHS Trust (n = 16) and Oxford University Hospitals NHS Foundation Trust (n = 20) in the UK.

105 participations had been recruited, but nine were excluded because of missing or invalid data and a further nine because of a protocol violation (they had already performed more than 10 fiberoptic intubations). Finally, one participant had been recruited twice: only the first of those attempts was kept for analysis.

### Demographics and pre-test dexterity

Table 1 shows the demographic data and Table 2 shows the pre-text dexterity. There was no difference in experience level with the fiberoptic bronchoscope or video game expertise between groups N and D and there was no difference in pre-test dexterity between the two groups.

**Table 1:** Demographic data.

	Total population	Group N	Group D	P-value^a^
	(n = 86)	(n = 43)	(n = 43)	
**Age (years)**	26 (25, 31)	26 (25, 30)	26 (25, 31)	0.47
**Gender**				
Male	51 (59.3%)	24 (55.8%)	27 (62.8%)	0.66
Female	35 (40.7%)	19 (44.2%)	16 (37.2%)	
**Dominant hand**				
Left-handed	9 (10.5%)	4 (9.3%)	5 (11.6%)	0.57
Right-handed	75 (87.2%)	37 (86%)	38 (88.4%)	
Ambidextrous	2 (2.3%)	2 (4.7%)	0 (0%)	
**Years of residency training**				
First year resident	43 (50%)	20 (46.5%)	23 (53.5%)	0.67
> First year resident	42 (48.8%)	22 (51.2%)	20 (46.5%)	
Other	1 (1.2%)	1 (2.3%)	0 (0%)	
**Previous fiberoptic experience**	65 (75.6%)	31 (72.1%)	34 (79.1%)	0.62
**Number of previous fiberoptic manipulations**				
0	21 (24.4%)	12 (27.9%)	9 (20.9%)	0.09
1–5	52 (60.5%)	28 (65.1%)	24 (55.8%)	
6–10	13 (15.1%)	3 (7%)	10 (23.3%)	
**Number of previous fiberoptic manipulations in the last 12 months**				
0	21 (24.4%)	12 (27.9%)	9 (20.9%)	0.62
1–5	65 (75.6%)	31 (72.1%)	34 (79.1%)	
6–10	0 (0%)	0 (0%)	0 (0%)	
**Estimated expertise level (0 to 10)**	1 (0, 2)	1 (0, 2)	1 (0, 2.5)	0.78
**Previous ORSIM**^**®**^ **experience**	14 (16.3%)	7 (16.3%)	7 (16.3%)	1
**Fiberoptic intubation on mannequin**	34 (39.5%)	16 (37.2%)	18 (41.9%)	0.82
**Video game experience**	57 (66.3%)	28 (65.1%)	29 (67.4%)	1
**Estimated video game expertise level (0 to 10)**	2,5 (0,6)	2 (0,6)	4 (1,6)	0.22
**Total number of scenario attempts during training**		12 (9,22)	4 (4,4)	
**Total training time** ^b^		15 (11,26)	14 (9,18)	0.08

Results expressed as median and interquartile range (1^st^ and 3^rd^ quartile) or as number and percentage.

^a^Difference between N and D using chi-square test or Fisher’s exact test for percentages, or Mann-Whitney test for ordinal variables.

^b^Raw training time data for Group D only measured times for each scenario, whereas for Group N only the total time for the entire training scession were available. In order to compare the training times between groups, 10 seconds were subtracted for each scenario reset/reload from the total training time for Group N

**Table 2:** Pre-test dexterity.

	Total population	Group N	Group D	P-value^a^
	(n = 86)	(n = 43)	(n = 43)	
**ORSIM**^**®**^ **score (%) 1st session**	40 (34, 45)	41 (34, 48)	40 (32, 44)	0.36
**ORSIM**^**®**^ **score (%) 2nd session**	42 (32, 50)	44 (38, 53)	40 (30, 46)	0.064
**Difference in score**	4 (-7, 13)	6 (-4, 13)	0 (-10, 10)	0.22

Results expressed as median and interquartile range (1^st^ and 3^rd^ quartile).

^a^Difference between group N and D using Mann-Whitney test.

### Final ORSIM^®^ score (primary outcome)

Table 3 shows the comparison of both groups ORSIM^®^ scores during the final testing (primary outcome). The final test ORSIM^®^ score for the normal airway scenario was significantly higher for group N than group D: median score 76% (IQR 56.5–90) versus 58% (IQR 51.5–69) respectively (p = 0.0039). There was no statistically significant difference between the two groups for the final test ORSIM^®^ score on any of the difficult intubation scenarios (Table 3).

**Table 3:** ORSIM^®^ scores’ (%) comparison between groups during final testing.

	Group N	Group D	P-value^a^
	(n = 43)	(n = 43)	
**Normal airways**	76 (56.5, 90)	58 (51.5, 69)	0.0039
**Airway trauma**	60 (44, 78)	58 (45, 70.5)	0.520
**Severe epiglottitis**	43 (22, 59.5)	52 (17, 68)	0.520
**False vocal cord cyst**	0 (0, 16.5)	2 (0, 28.5)	0.284
**Angioneurotic oedema**	39 (16.5, 51)	40 (14.5, 59.5)	0.832

Results expressed as median and interquartile range (1^st^ and 3^rd^ quartile).

^a^Difference between group N and D using Mann-Whitney test.

### Performance evolution on normal airways

During the first try, at the beginning of training, there was no difference between groups in their performance on the normal airway scenario for ORSIM^®^ scores, time to succeed and collision avoidance. During the final testing on the normal airway scenario (after completion of the training) the ORSIM^®^ score for group N was significantly higher and their times and estimated level of difficulty were significantly decreased when compared to Group D (Table 4).

**Table 4:** Performance evolution on normal airway scenario between first try and final testing according to groups.

	Total population	Group N	Group D	P-value^a^
	(n = 86)	(n = 43)	(n = 43)	
**First try characteristics**				
ORSIM^®^ score (%)	52 (46, 66.8)	50(46, 68)	53 (45.5, 66)	0.78
Time (in seconds)	44.5 (29.2, 69.8)	45 (29, 72)	42 (30.5, 61)	0.42
Collision avoidance (%)	99 (80.2,100)	100 (79, 100)	91 (81, 100)	0.65
Perceived level of difficulty (0–100)	38 (17.2, 50.8)	50 (39, 61.5)	22 (12, 37)	<0.0001
**Difference between first try and final testing**				
ORSIM^®^ score (%) change	8 (0.2, 20)	16 (7,23)	4 (-1.5, 9.5)	<0.0001
Change in time (in seconds)	-12 (-27.8,-3)	-18 (-37, -8.5)	-6 (-18, -0.5)	0.0018
Change in collisions avoidance (%)	0 (-4.5, 9.2)	0 (0,11.5)	0 (-6.5, 8.5)	0.199
Change in perceived level of difficulty	-14 (-32.8,-4)	-29 (-45,-11.5)	-7 (-17.5, 0.5)	<0.0001

Results expressed as median and interquartile range (1^st^ and 3^rd^ quartile).

^a^Difference between group N and D using Mann-Whitney test.

### Comparison of perceived level of difficulty between groups during final testing

There was a significant difference of perceived level of difficulty on a VAS (0 to 100) between the two groups concerning the severe epiglottitis scenario as it appeared easier to group D than to group N: 66 (57–80) versus 79 (68–90) for the severe epiglottitis, p<0.05) (Table 5).

**Table 5:** Comparison of perceived level of difficulty for each scenario according to groups during final testing (Visual Analogic scale (VAS) from 0 to 100).

	Group N	Group D	P-value^a^
**Normal airways**	*(n = 43)* 12 (8, 26)	*(n = 43)* 12 (8, 17.5)	0.180
**Airway trauma**	*(n = 39)* 73 (65.5, 83)	*(n = 40)* 66 (58, 78.5)	0.091
**Severe epiglottitis**	*(n = 41)* 79 (68, 90)	*(n = 41)* 66 (57, 80)	0.0052
**False vocal cord cyst**	*(n = 18)* 86 (71.5, 91.5)	*(n = 24)* 83.5 (60.8, 94.2)	0.939
**Angioneurotic oedema**	*(n = 40)* 81.5 (66.8, 97)	*(n = 39)* 72 (64, 83.5)	0.091

Results expressed as median and interquartile range (1^st^ and 3^rd^ quartile).

Only participants who succeeded in the scenario were evaluated.

^a^Difference between group N and D using Mann-Whitney test.

## Discussion

This study shows a significant performance improvement for fiberoptic intubations on normal airways for the group who trained only on normal airways. In contrast, training on a variety of difficult airways did not translate to performance improvement on (other) difficult airway scenarios or on a normal airway.

Simulation appears as a new tool allowing learners to achieve a satisfactory skill level before practice. Graeser *et al*. [26] showed that simulator training allows for entry of the learning curve of airway management at a higher level. J.E Smith *et al*. [33] described a learning curve of 10 fiberoptic intubations to achieve a satisfactory competence and 30–50 fiberoptic intubations to reach expert level. Repetition of a same scenario seems to be the main determinant of learning. In our study, participants in group N had performed a median of 12 successive normal intubations before being tested (over a median of 15 minutes, Table 1), compared to only one single attempt in group D. In group D, each scenario could only be done once and participants in group D had thus performed the balance of their training on various difficult airways (over a median total of 14 minutes, Table 1). As our study shows, that training did not translate into better performance on the normal airway scenario.

In their study on validating the ORSIM^®^ [30], Baker *et al*. proposed a score of 70 to characterize an expert level. Our study shows a median ORSIM^®^ score of 76% after the 12 attempts in the final testing phase for group N on the normal airway, suggesting an satisfactory competence, or even expert level had been acquired after their repetitive training with this same normal airway scenario. On the other hand, for group D, the median ORSIM^®^ score for each scenario remained well below 70%. It would be interesting to see if the ORSIM^®^ score during final testing for group D would have improved significantly if Group D also would have trained until achieving ‘expert level’ (score of at least 70%) on each of their difficult training scenarios, but our experimental design did not allow to study that (and would have likely led to group D training for a much longer total time compared to group N, thus introducing a confounder).

The main finding of our study is thus that the learning may initially be limited to the actual scenario and the degree with which learning extends to other scenarios remains unclear (but clinically important). It may be necessary to introduce repetitive training sessions of each difficult scenario before the full benefits would become apparent.

Although training on a variety of difficult airways for group D did not translate to performance improvement, it did seem to lead to a reduction of perceived difficulty level some scenarios (for the severe epiglottitis scenario). Similarly, acquisition of expertise level for group N significantly reduced the perception of difficulty on the normal airway scenario. In practice, we can often observe that stress and perception of difficulty can have a negative impact on performance—and training is probably the only way to remedy this [34]. Our study shows that training on difficult airway scenarios could indeed reduce the stress during subsequent attempts on difficult airway scenarios.

Yang *et al*. [35] showed that psychometric skills can predict the acquisition of procedural skill performance and Louridas et al. [36], looking at laparoscopic surgery, investigated the value of psychometric testing to predict the technical performance of new residents. It would be interesting to study the relationship between dexterity and time to reach an expert level for fiberoptic intubation skills for a ‘competence by design’ approach to teach fiberoptic intubation.

### Limitations of the study

Due to the unforeseen circumstances posed by COVID-19 we were forced to halt trial recruitment and decided to perform an unplanned interim analysis of the results. We put in place a plan for a single interim analysis with a symmetrical stopping rule for a p< 0.002 using the Pocock rule. When we identified a p = 0.0039 for our primary outcome in the direction of improved outcomes for the normal group we decided to stop the trial based on futility. While the number of participants in our study was smaller than planned, the magnitude of the estimated effect sizes was consistently small. Furthermore, the group trained on the difficult scenarios were as likely to show worse results as they were to show improved results. For these two reasons we postulate that the inclusion of more participants would not change the primary outcome.

Some participants in both groups had already used the ORSIM^®^ simulator, however the numbers were small and also well distributed across both study groups, thus unlikely to create a bias. While it was originally contemplated to exclude participants with previous ORSIM^®^ experience, it was felt that this would unnecessarily restrict the recruitment in view of the fact that the use of ORSIM^®^ simulation was expanding rapidly.

In our study, we only assessed the level 2 of Kirkpatrick’s model of training evaluation: the testing of skills based on training [37]. It will be important to investigate actual changes in clinical practice (level 3) and ultimately changes in patient outcome (level 4) when evaluating the role of the ORSIM^®^ simulator. The latter steps present significant logistical and ethical challenges and may involve the creation of actual patient airway through 3 D printer technology as an interim step.

## Conclusion

This study was not able to demonstrate a benefit of adding difficult airway scenarios for learner’s performance during intubation training with a replica flexible bronchoscope. It only showed that repeated training on the ORSIM^®^ simulator with the same scenario (a normal airway) led to increased intubation skills for the normal airway and that this skill was specific for the normal airway scenario. Training on normal airways did not predict better skills on difficult airways (and vice versa).

Subsequent studies should focus on the acquisition of a level of expertise in difficult airways and its reproducibility from one difficult airway scenario to another in order to validate this simulation model and better prepare the learner for clinical practice.
